# Current Concepts on the Pathogenesis of Systemic Sclerosis

**DOI:** 10.1007/s12016-021-08889-8

**Published:** 2021-09-06

**Authors:** Marie Elise Truchetet, Nicolò C. Brembilla, Carlo Chizzolini

**Affiliations:** 1 ImmunoConcEpt, CNRS, UMR 5164, University of Bordeaux, Bordeaux, France; 2 Rheumatology Department, CHU Bordeaux Hospital, Bordeaux, France; 3 Pathology and Immunology, School of Medicine, University Medical Center, Geneva, Switzerland

**Keywords:** Systemic sclerosis, Pathogenesis, Immune responses, Fibrosis, Inflammation, System biology

## Abstract

From the clinical standpoint, systemic sclerosis (SSc) is characterized by skin and internal organ fibrosis, diffuse fibroproliferative vascular modifications, and autoimmunity. Clinical presentation and course are highly heterogenous and life expectancy variably affected mostly dependent on lung and heart involvement. SSc touches more women than men with differences in disease severity and environmental exposure. Pathogenetic events originate from altered homeostasis favored by genetic predisposition, environmental cues and a variety of endogenous and exogenous triggers. Epigenetic modifications modulate SSc pathogenesis which strikingly associate profound immune-inflammatory dysregulation, abnormal endothelial cell behavior, and cell trans-differentiation into myofibroblasts. SSc myofibroblasts show enhanced survival and enhanced extracellular matrix deposition presenting altered structure and altered physicochemical properties. Additional cell types of likely pathogenic importance are pericytes, platelets, and keratinocytes in conjunction with their relationship with vessel wall cells and fibroblasts. In SSc, the profibrotic milieu is favored by cell signaling initiated in the one hand by transforming growth factor-beta and related cytokines and in the other hand by innate and adaptive type 2 immune responses. Radical oxygen species and invariant receptors sensing danger participate to altered cell behavior. Conventional and SSc-specific T cell subsets modulate both fibroblasts as well as endothelial cell dysfunction. Beside autoantibodies directed against ubiquitous antigens important for enhanced clinical classification, antigen-specific agonistic autoantibodies may have a pathogenic role. Recent studies based on single-cell RNAseq and multi-omics approaches are revealing unforeseen heterogeneity in SSc cell differentiation and functional states. Advances in system biology applied to the wealth of data generated by unbiased screening are allowing to subgroup patients based on distinct pathogenic mechanisms. Deciphering heterogeneity in pathogenic mechanisms will pave the way to highly needed personalized therapeutic approaches.

## Introduction

Systemic sclerosis (SSc) is clinically characterized in the one hand by fibrosis of skin and internal organs leading to altered organ structure and ultimately organ dysfunction and on the other hand by functional and structural vasculopathy resulting among others in Raynaud phenomenon, digital ulcers, pulmonary artery hypertension, and renal crisis [1]. In SSc, fibrosis and vasculopathy are intimately associated and lead to highly heterogeneous clinical manifestations with a widely variable prognosis. Main causes of death are lung and heart involvement which may occur early or late in the disease course [2]. Standardized mortality rates range from 2.82 to 3.64 in the most recent meta-analysis [3]. In addition, SSc imposes high burden in terms of quality of life and social cost.

Inflammation is the physiological response to altered tissue and organ homeostasis and is the common denominator to SSc pathogenesis. We believe that inflammatory processes are keys to initiation and progression toward both fibrosis and structural vasculopathy in response to events perturbing homeostasis. However, deciphering the multiple components of inflammation, which simultaneously act in many different, often opposing, directions remains an important aim to understand SSc pathophysiology.

**Fig. 1:**
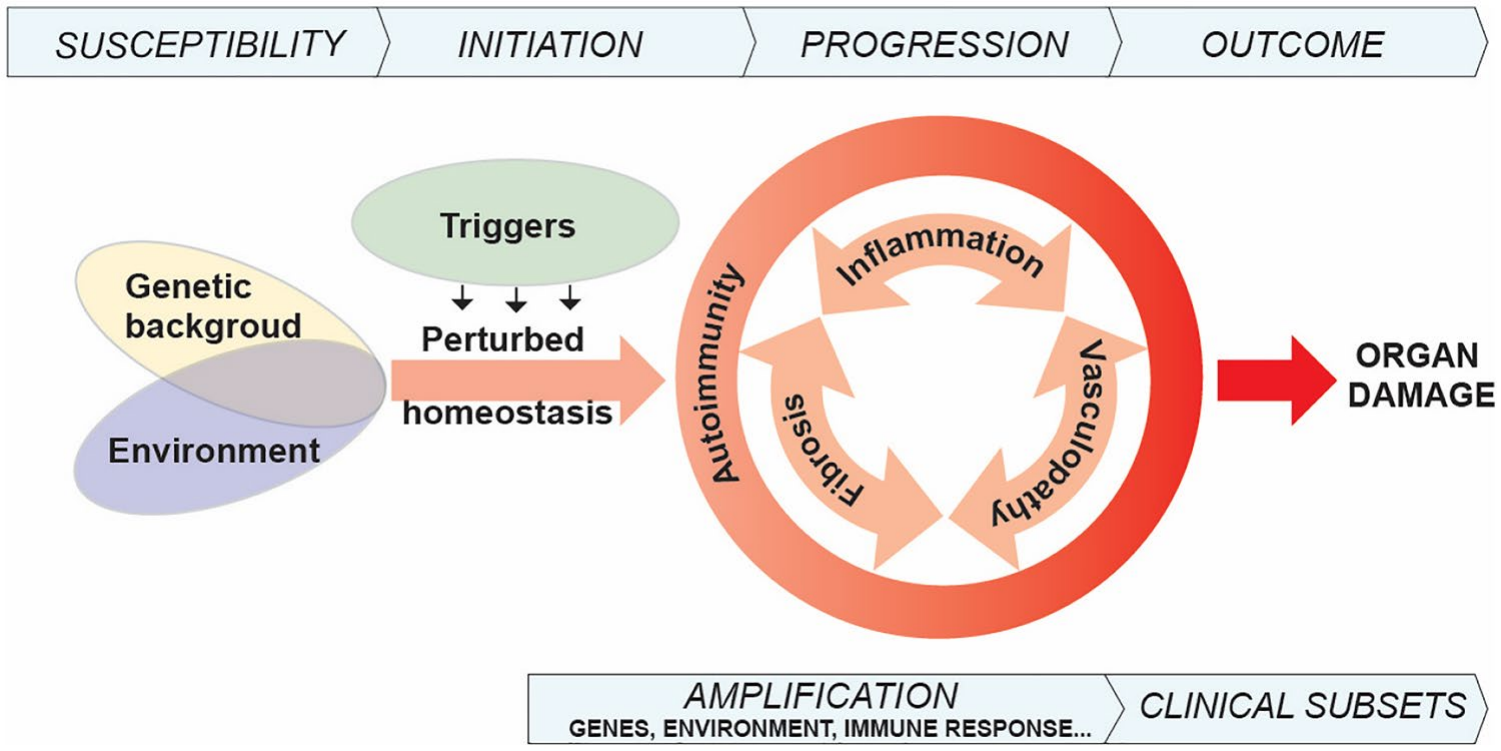
Overview of conditions and events leading to systemic sclerosis. Schematic diagram highlighting the complex interplay thought to play a role in susceptibility and initiation of SSc in which genetic predisposition and environmental cues under the pressure of a variety of triggers lead to perturbed homeostasis with ensuing autoimmunity. Autoimmunity is represented as the common denominator of the three fundamental aspects of SSc: inflammation, vasculopathy, and fibrosis. Heterogeneous clinical manifestations would then develop according to variable amplification mechanisms resulting in recognized clinical subsets and organ damage

From the pathogenic point of view, the questions to be answered are many and should address the predisposing genetic background, the trigger(s) as well as the mechanisms involved in the initiation, and further development of both fibrosis and vasculopathy simultaneously taking into account clinical heterogeneity (Fig. 1). The term of “intermediate pathophenotypes” has been proposed by C. Feghali-Bostwick and J. Varga to accommodate the dynamic processes underlying heterogeneity in SSc and our understanding of the mechanisms involved in SSc pathogenesis at cellular and tissue levels.

Excellent reviews have been recently published addressing various aspects of SSc pathogenesis [4–13]. Here, we will attempt to provide a synthetic view of the main aspects of SSc pathogenesis.

### SSc Disease State

**Fig. 2:**
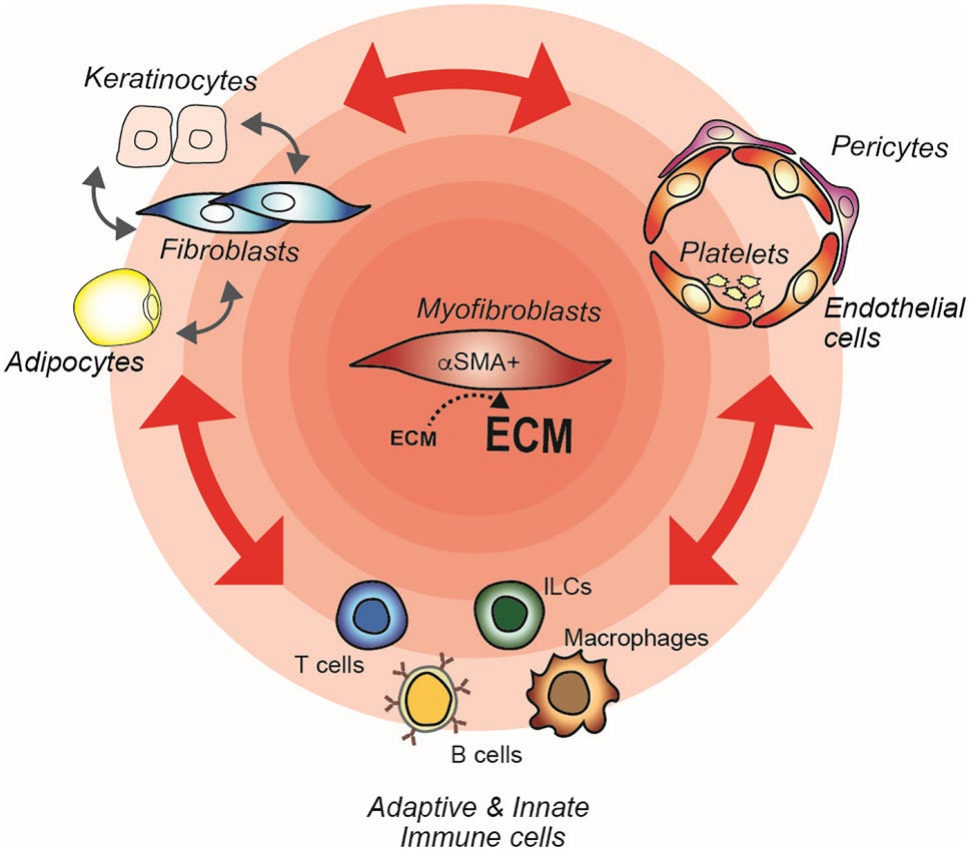
Major cell types and their multiple interactions in SSc pathogenesis. SSc is here viewed as a three-leg pathology in which major dysfunctional cell types are immune cells, endothelial cells, and fibroblasts which directly or indirectly intensely interact leading to myofibroblast hyperactivation. This cell and soluble factor three-leg network establishes further interactions with many other cell types including adipocytes, keratinocytes, pericytes, and platelets. The concentric reddish shadow highlights the influence of the various cell types on the activation of myofibroblasts. Two-head red arrows indicate multiple, reciprocal interactions mainly ensured by soluble mediators of inflammation. Dashed arrow indicates increase in extracellular matrix (ECM) deposition by myofibroblasts

The most commonly postulated model of disease progression in SSc is sequential, with immune activation and subsequent vasculopathy leading to activation of fibroblasts and fibrosis as the end effect of these processes. However, substantial debate animates the SSc community on what order these events take place. According to the definition provided by Stern and Denton, the disease state is only tolerated if there is simultaneous dysregulation of the immune system, vascular endothelium, and connective tissue repair system [6]. Thus, SSc can be viewed as a three-leg pathology in which major dysfunctional cell types are immune cells, endothelial cells, and fibroblasts which intensely interact mostly via soluble mediators directly or indirectly leading to myo-fibroblast hyperactivation. This cell and soluble factor three-leg network establishes further interactions with many other cell types of which keratinocytes, pericytes, platelets, and adipocytes have attracted particular attention in recent years (Fig. 2).

## SSc Genetic Background

### Family Studies

 Compared to the general population, the risk of developing SSc is higher in first-degree relatives of persons suffering of SSc and strong family clustering, with an estimated risk of 1.6% versus a 0.026% [14]. However, the concordance rate for clinical disease in twins is relatively low (4.7% in one study) with higher frequency in concordance for the presence of autoantibodies and T cell responses irrespective of clinical expression [14–16]. This is strong evidence for the heritability of SSc, at the same time indicating a weak association with disease phenotype.

### HLA

Systemic autoimmunity is favored by a genetic background in which genes and gene polymorphisms associated with the major histopathologic complex (MHC) or human leucocyte antigens (HLAs) are of major importance. This is the case also for SSc, but most interestingly, the associations between HLA haplotypes and SSc vary according to ethnicity and autoantibody (autoAb) status. Thus, risk alleles may be different in Fareast Asia compared to Europe or America and within USA according to ethnic origin. For example, in European Americans (EA) and Latino Americans (LA), the DRB1*1104, DQA1*0501, DQB1*0301 haplotype, and DQB1 alleles encoding a non-leucine residue at position 26 (DQB1 26 epi) showed the strongest associations with SSc, while the strongest association for African Americans (AA) was with DRB1*0804 and HLA-DRB1*1102. DRB1*0804, DQA1*0501, DQB1*0301, and DPB1*1301 alleles showed the highest odds ratio for anti-topoisomerase autoAb (ATA) (OR = 14) and HLA-DRB1*0804 for antifibrillarin autoAb (AFA) (odds ratio = 7.4) in AA. The anti-centromere autoAb (ACA) were best explained by DQB1*0501 and DQB1*26 epi alleles and anti-RNA polymerase autoAb (ARA) by DRB1*0404, DRB1*11, and DQB1*03 alleles in EA and LA subjects. Nonetheless, HLA-DPB1*1301 allele was associated with the ATA+ in both AA and EA patients demonstrating a transancestry effect [17, 18].

### GWAS

In addition to the impact of HLA genes, candidate gene approaches and more substantially genome-wide association studies (GWAS) by assessing SNP (single nucleotide polymorphism) associations provided evidence on the contribution of chromosomal locations to the risk of developing SSc. Interestingly, most of the identified genetic regions which polymorphisms are associated with SSc involve intronic or intergenic regions. Recent evidence suggests that these regions may have regulatory function by interacting with gene promoters or enhancers. A recent collaborative effort, by applying a meta-analysis on 14 independent European cohorts comprising a total of 26,679 individuals (9095 SSc patients and 17,584 healthy controls) has identified 23 genomic regions significantly associated with SSc of which 12 most likely causal [19]. Interestingly, these authors identified 43 robust target genes of these regions, thus showing that the expression of more than one gene is influenced by these polymorphisms. Remarkably, the majority of recognized polymorphisms are relevant for the immune response particularly associated with five main molecular pathways identified by in silico analysis: (a) IFN-I signaling pathway, (b) T cell activation, (c) B cell activation, (d) NFkB pathway, and (e) immune system process. However, additional susceptibility genes are relevant for endothelial cells therefore potentially associated with vasculopathy, and fibroblasts with fibrosis. Table 1 inspired from [20] reports enriched SSc risk genes, their function, and cells likely involved.

**Table 1:** Non-HLA risk genes associated with SSc

**Risk genes**	**Name/function**	**Characteristic**	**Main cell target**
*DDX6*	Mediates mRNA degradation	Hypoxia decreases DDX6 expression	EC
*GRB10*	Cell growth inhibitor		EC
*SOX5*	Transcription factor	Cell fate determination	F
*CSK*	c-Src thyrosine kinase	Regulates differentiation	F
*CAV1*	Caveolin-1	Induces TGFβ-R internalization/degradation	F
*DNASE1L3*	DNA fragmentation during apoptosis NET degradation		Many
*TNFAIP3*	Signaling inhibitor (also named A20)	NFκB pathway	Many
*TNIP1*	Signaling inhibitor	NFκB pathway	Many
*IRF5*	IFN-I signaling	Associated with ILD and dSSc	DC; EC; F; Mϕ;
*IRF7*	IFN-I signaling		DC; EC; F; Mϕ;
*IRF8*	IFN-I signaling		DC; EC; F; Mϕ; T cells
*TLR2*	PAMP sensing	Increased production of IL-6 by DC	DC, Mϕ, F
*TNFSF4*	Encodes OX40L	Co-stimulatory	DC
*GSDMA/B*	Gasdermin / pyroptosis	Inflammatory cell death	Mϕ
*RAB2A*	Autophagosome clearance	May impair autophagy	EC; Mϕ
*ATG5*	Autophagy, many roles		DC, EC, Mϕ
*BANK1*	Involved in B cell activation	B cell responses	B cells
*BLK*	Src Family Tyrosine Kinase	B cell biology	B cells
*PRDM1*	Transcription factor /BLIMP1	Plays a role in innate and adaptive immune cells	NK; T cells, B cells
*CD247*	tzeta subunit TcR	T cell activation	T cells
*STAT4*	Signal transducer, transcription factor	Phosphorylated in response to IFN, IL-12, IL-23	T cells
*PTPN22*	Thyrosine phosphatase	TcR signaling, decreased function	T cells
*CCR6*	Chemokine receptor	Recruite Th17 cells	T cells
*IL21*	Interleukin	Th follicular	T cells
*IL12RB1*	IL-12 receptor chain	T cell polarization	T cells, NK cells
*IL12RB2*	IL-12 receptor chain	T cell polarization	T cells, NK cells
*SCHIP1-IL12A*	Intergenic	IL-12 polarizes Th1 cells	T cells, NK cells

*DC* dendritic cell, *dSSc* diffuse cutaneous systemic sclerosis, *EC* endothelial cell, *F* fibroblast, *IFN* interferon, *IL* interleukin, *ILD* interstitial lung disease, *M*ϕ macrophage, *NF*κ*B* nuclear factor “kappa-light-chain-enhancer” of activated B-cells, *TcR* T cell receptor, *TGF*β transforming growth factor-beta, *Th* T helper cell

### Epigenetic Regulation

Substantial differences have been demonstrated in the epigenetic tags when SSc were compared to healthy fibroblasts. In one study, hypomethylated genes included ITGA9, encoding an α integrin and other relevant genes such as ADAM12, COL23A1, COL4A2, and MYO1E, and transcription factors genes RUNX1, RUNX2, and RUNX3 were hypomethylated in both dSSc and lSSc. Pathway analysis of differentially methylated genes in both dSSc and lSSc revealed enrichment of genes involved in extracellular matrix-receptor interaction and focal adhesion [21]. Another study focusing on Wnt signaling in mononuclear cells and fibroblasts found that the promoters of DKK1 (Dickkopf WNT signaling pathway inhibitor 1) and SFRP1 (secreted frizzled-related protein 1) were hypermethylated in SSc. Promoter hypermethylation resulted in impaired transcription and decreased expression of DKK1 and SFRP1 in SSc [22]. Since DKK1 is an inhibitor of the Wnt/β-catenin signaling cascade which deeply involved in fibrosis development, decreased DKK1 expression may account for greater pro-fibrotic signaling.

Gene transcription is also regulated by miRNA, of which some have been associated with SSc; miR-21 and miR-155 appear to have profibrotic properties, while let-7 and miR-29 are rather profibrotic. In addition, a significant decrease in the levels of miR-29 has been found in lesional SSc skin [23]. Increased expression of miR-92a was reported in SSc fibroblasts resulting in reduced MMP-1 expression [24].

## SSc Triggers

Within genetic susceptibility, many triggers may be involved in disease initiation. They may operate sequentially and manifest gender preferences.

### Chemicals

The association between environmental risk factors and SSc has been extensively analyzed, but the role of the environment is not yet fully understood [25, 26]. Environmental factors can be classified as occupational (silica, organic solvents) and non-occupational/non-infectious (drugs, pesticides, silicones, heavy metals) [25, 27]. According to a recent meta-analysis, the strongest evidence indicates that silica and organic solvents are risk factors for SSc. Exposure to vinyl chloride, white spirit, solvents, crystalline silica among others, and use of tryptophane have been associated with SSc or SSc-like disorders [28]. While there is substantial evidence that exposure to silicones is not a risk factor for SSc, the meta-analysis of breast implants exposure highlighted a slight over-risk [overall OR 1.68 (95%CI 1.65–1.71; *p* < 0.001)]. The risk of SSc following exposure to silica is higher in males compared with females with more frequent diffuse cutaneous SSc and lower survival rates [26, 29].

### Infectious Agents

Infectious agents may participate in breaking T and B cell tolerance by molecular mimicry and by the simultaneous activation of innate responses when pathogen-associated molecular patterns (PAMPs) activate pattern recognition receptors (PRRs), thus tuning the immune system to enhanced responses. Immune effector mechanisms may then participate to cell damage. Parvovirus B19, cytomegalovirus (CMV), Epstein–Barr virus (EBV), and retroviruses have all been proposed as initiating triggers of SSc [30, 31]. Particular attention has been attracted by CMV which genetic material has been found in endothelial cells and suspected to elicit IgG that specifically recognized the CMV late protein UL94 and the endothelial cell surface integrin–NAG-2 protein complex, thereby inducing endothelial cell apoptosis [32]. These IgG may also activate fibroblasts and enhance collagen production [33]. The presence of Parvovirus B19 DNA in the bone marrow and/or skin biopsies has been reported. By in situ RT-PCR, the presence of Parvovirus B19 DNA and TNF was demonstrated in endothelium and fibroblasts [34]. While not replicating in fibroblasts, Parvovirus B19 can activate many genes involved in inflammation and fibrosis [35]. Similarly, EBV infection was shown to induce aberrant toll-like receptor (TLR) activation pathway and fibroblast-myofibroblast conversion in scleroderma [36]. From a different angle, when bioinformatically predicting the T cell immunodominant peptides of topoisomerase 1, fibrillarin, and centromere protein A in association with selected HLA α/β allelic heterodimers, it was reported that these autoantigens are homologous to viral protein sequences from the *Mimiviridae* and *Phycodnaviridae* families. These data suggest a possible link between HLA alleles, autoantibodies, and infectious triggers in the pathogenesis of SSc [18].

### Neoplastic Diseases

SSc has complex relationships with many different types of cancer [9]. A close temporal association between the onset of SSc and the detection of cancer has been described in a subset of patients positive for anti-RNA polymerase III (RNApol III) antibodies [37]. This observation led to the discovery that mutated autoantigens (RNApol3) are present in the tumors obtained from these patients and result in mutant-specific T cell immune responses as well as in the generation cross-reactive autoantibodies [38]. These findings support the possibility that, at least in some patients, an abnormal (mutated) cancer antigen may be the initial trigger for an autoimmune T cell activation in SSc and autoAb recognizing the mutated RNApol III, which then cross-react with the wild-type autoantigen.

### Microchimerism

Feto-maternal microchimerism, which is the transplacental passage of semi-allogenic fetal cells to the mother or vice versa the passage of semi-allogenic maternal cells to the fetus, may trigger autoimmunity in SSc [39]. It is supposed that microchimeric cells may provide chronic stimulation due to MHC-mismatch with enhanced expansion of alloreactive, profibrotic Th2 cells [40]. Exposure to vinyl chloride may enhance the pathogenic role of microchimeric cells in murine models [41], an interesting example of the combined effect of multiple triggers operating in conjunction or sequentially to favor SSc.

## Sex Bias in SSc

As many other systemic autoimmune disorders, SSc preferentially affects women with a female to male ratio exceeding 4 to 1 [42–44]. Substantial differences in the clinical presentation and environmental exposure underlie gender differences in SSc. Thus, at diagnosis men preferentially present an active and diffuse form of the disease with more frequent heart and lung involvement which may impact on survival [45 46]. Exposure to chemicals is more frequent in males [28, 43], suggesting that perturbed homeostasis by environmental cues substantially adds to pathogenetic mechanisms which are enhanced in females.

### Sex Hormones

Sex hormones and their cyclic variation during the fertile years have profound impact on the immune response and likely they play a role on female preponderance in SSc. Broadly speaking, estrogens tend to enhance the adaptive immune responses and in particular the production of (auto)-antibodies, while progesterone and androgens may exert inhibitory functions [47–49]. A recent systematic review of the literature conducted on the role of sex hormones in SSc reported that estrogens may be simultaneously fibrogenic and vasodilatory. Within the limitation of the small numbers of individuals studied, compared to healthy controls women with SSc tended to have lower levels of androgens, non-significantly higher levels of estradiol, while men had increased levels of estradiol [50].

### X-chromosome

The large excess in genes present in the X-chromosome compared to the Y-chromosome is compensated by the inactivation of one X-chromosome (XCI) copy of the two present in females. This is a random and active process implicating the long non-coding RNA named *XIST*, which silences by epigenetic modifications almost all genes present in X-chromosome [51]. In females, escape from XCI may thus allow the expression of two copies of the genes encoded in the X-chromosomes, of which many are relevant for the immune response and for which the escape from inactivation has been demonstrated [52]. For instance, the expression of two copies of TLR7 in B cells of healthy females was shown to result in higher production of antibodies [53]. The relevance in SSc of such a mechanism is currently being explored. Enhanced X monosomy in SSc women [54] and specific patterns of X chromosome gene methylation in peripheral lymphocytes from monozygotic twins discordant for scleroderma [55] have been demonstrated. Intriguingly, extreme bias in XCI has been shown in SSc and correlated to a decreased expression of *FOXP3* and reduced Treg function [56]. Further, single nucleotide polymorphisms (SNPs) enriched in SSc have been identified in X chromosome genes involved in the immune response such as *IL13RA2*, *IRAK1*, and *FOXP3*, and while this has not formally being proven, these SNPs may contribute to SSc development in females [57–60]. About 10% of miRNAs are located on X-chromosome and may escape inactivation or be subjected to skewed X inactivation; therefore, they may also participate in gender-related differences in SSc pathogenic mechanisms [61].

## SSc Initial Events

The question of what is first in SSc pathogenesis has no definitive answer and spurs substantial debate. From the clinical stand point, Raynaud phenomenon in the large majority of cases initiate months to years before other clinical manifestations become apparent, including skin and organ fibrosis. Based on this chronological clinical order, many authors suspect that vasculopathy is the initiating event. In this perspective repeated vasospastic episodes triggered by cold exposure occurring in the appropriate genetic background may result in altered homeostasis in relationship to ischemia/reperfusion processes and may provide the substrate for inflammatory responses and structural vasculopathy. Endothelial cell injury is proposed as a crucial initiating event leading to vascular remodeling with intimal proliferation of arterioles and capillary breakdown and finally, blood vessel occlusion [7, 62, 63]. Of note, recognized mechanisms leading to endothelial cell injury are mostly immunologic in nature. Anti-endothelial cell autoantibodies through ADCC, anti-endothelin, and anti-angiotensin agonistic antibodies, cytolytic CD4+ T cells, γ/δ T cells, and NK cells have been described as effector of endothelial cell (EC) activation and/or damage [7, 64–67] If this is true, then vasculopathy follows innate and adaptive immune responses (Fig. 1). Consistently with this kinetic, the presence of serum anti-nuclear antibodies—evidence for adaptive immune responses—detected at first evaluation of Raynaud’s is considered an important, independent predictive element to classify Raynaud as secondary to SSc [68, 69]. Taken from a different perspective, it is known that monocyte/macrophage and T cell inflammatory perivascular infiltrates are detectable early in SSc [70] and ultrastructural EC damage appears to follow the appearance of inflammatory mononuclear infiltrates [71]. Thus, intricate mechanisms are at play in early events leading to SSc in which components of the immune response in relationship with EC and vessel function and integrity play a role, well before fibrosis initiate developing.

## SSc Vasculopathy

**Fig. 3:**
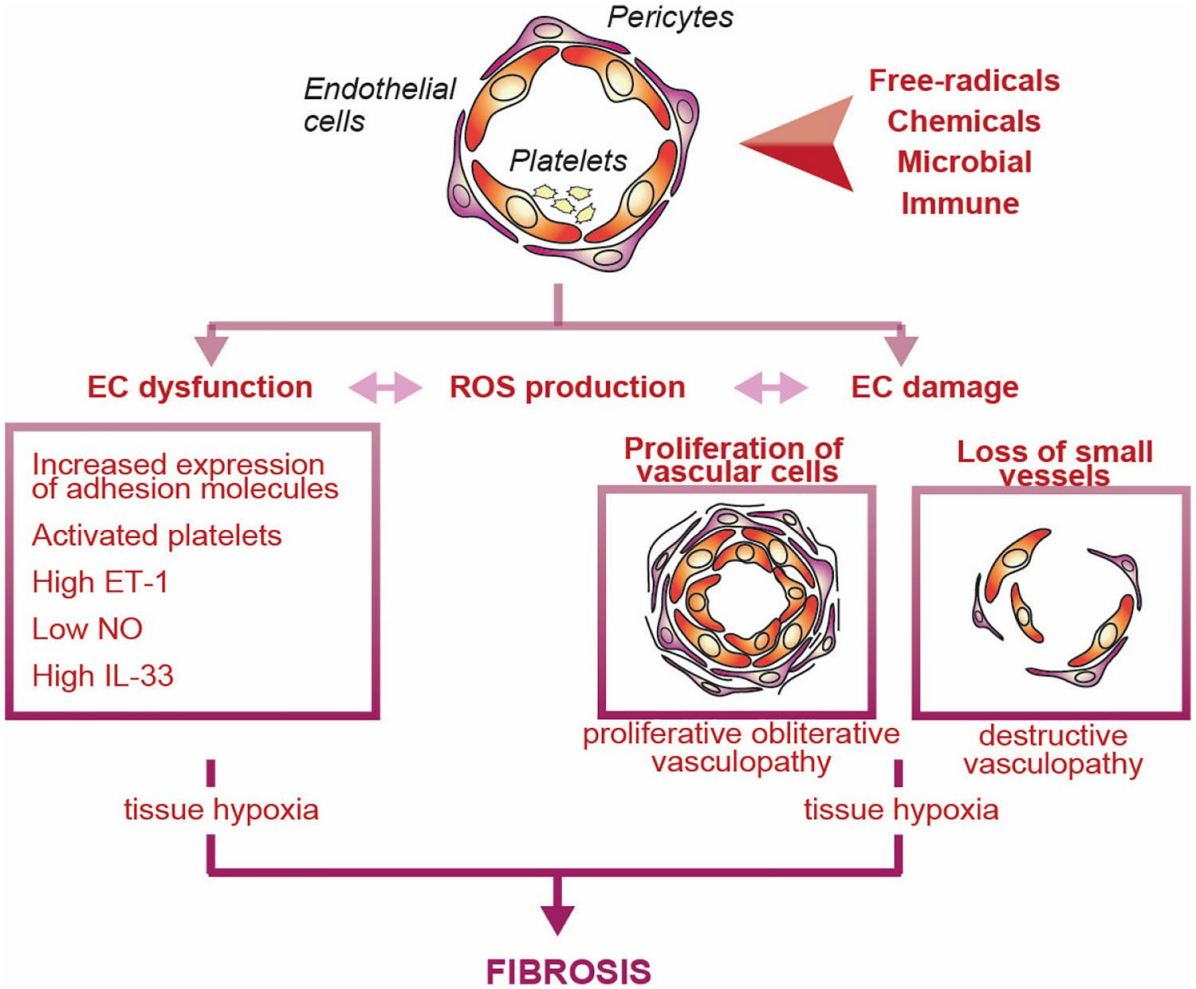
Vasculopathy in SSc. In SSc, under the influence of a variety of stimuli here depicted as a bicolor arrowhead, EC become dysfunctional and undergo damage. Excess in vasoconstricting over vasodilating agents, as well as enhanced fibroproliferative events of the vessel wall associated with reduced angiogenesis and vasculogenesis are characteristic. Vasculopathic alterations contribute to the developments of fibrosis. ET-1, endothelin-1; IL, interleukin; NO, nitric oxide; ROS, radical oxygen species

Fibroproliferative modifications of vessel walls and rarefaction of capillaries underpin vasculopathy in SSc which affects mainly the micro-circulation, but also the macro-circulation. Endothelial cell (EC) dysfunction and damage are considered cornerstones of SSc vasculopathy (Fig. 3). Indeed, structural damage and inappropriate repair events distinguish primary form secondary Raynaud. Initial mechanisms may involve selective increased expression of alpha 2 adrenergic receptors on vascular smooth muscle cells (vSMC) with increased response to catecholamines [72]. Imbalance between vasodilating and vasoconstricting agents with reduced production of nitric oxide (NO) and enhanced production of endothelin-1 (ET-1) may lead to ischemia / reperfusion and subsequent increased oxidative stress which impact on EC [73]. Platelet activation may participate by releasing potent vasoconstrictors such as thromboxane and serotonin [74]. Transition to inflammatory events then occurs with opening of tight EC junctions, fluid leakage in the extravascular space, and enhanced expression of adhesion molecules, all favoring the recruitment of mononuclear cells. EC injury may lead to EC apoptosis [75]. Infectious agents, autoantibodies, toxic compounds, and cytolytic T and NK cells may be causes of EC apoptosis. Significant intimal proliferation and accumulation of proteoglycans in the arterioles and small sized arteries are common in SSc [76]. Moreover, abnormality of the vessel wall is likely to result from increased synthesis of extracellular matrix (ECM) by intimal and adventitial fibroblasts. Transdifferentiation of EC via the process of endothelial-mesenchymal transition (EndoMT) and more likely of pericytes into profibrotic myofibroblasts may contribute further to vascular wall fibrosis [77]. On the other hand, vSMC, under the influence of hypoxia, cytokines and growth factors may migrate into the intima, differentiate, and then synthesize the matrix of the fibrotic vascular lesions.

Both defective angiogenesis (growth of new vessels from existing vessels) and vasculogenesis (*de novo* formation of new vessels) likely contribute to capillary rarefactions. Angiogenesis is disturbed through expression of inefficient pro-angiogenic mediators, upregulation of inhibitors of angiogenesis and by alteration of transcripts involved in signal transduction pathways [78]. Hypoxia enhances the production of VEGF which is detectable in high amount in SSc sera. However, the relative abundance of a non-signaling variant (VEGF165b) and alterations at the receptors level may contribute to altered angiogenesis [79]. Imbalance between other pro-angiogenic factors and their receptors are also at play [80]. Endothelial progenitor cells (EPCs) originating from the bone marrow are fundamental for vasculogenesis. Although discrepancies between various reports exist, possibly related to differences in the markers used for the identification of these progenitors, the number of circulating EPC appears to be reduced in SSc, which may contribute to defective vasculogenesis. Alternatively, their recruitment at lesional sites could be impaired as suggested by the relative lack of the recruitment factor cellular communication network factor-1(CCN1) reported in SSc digital ulcers [81].

EC may respond to and produce a variety of cytokines and other soluble products of inflammation. Thus, they may influence the behavior of resident or recently recruited cell types in the skin and other organs. Among many others, interleukin-1 (IL-1), thymic stromal lymphopoietin (TSLP) [82], and IL-33 appear to play important roles in the interaction with macrophages, other innate immune cells, fibroblasts, and adipocytes. IL-33, which levels are increased early in the SSc disease course, might mediate very early pathogenic events of SSc through recruitment and stimulation of cells expressing the appropriate receptor [83–86].

## Fibrosis and Fibroblasts in SSc

### Fibrosis

Fibrosis is the default inflammatory response to chronic tissue injury of whatever cause aiming at containing and circumscribing tissue damage. Fibrosis itself consist in the enhanced deposition over resorption of ECM. In fibrotic tissues, the ECM appears to be structurally altered. Fibrosis in SSc can be seen as a process resembling wound healing in which the resolution phase is not efficacious or even does not occur.

### The Pro-fibrotic Milieu

When examined in animal models, wound healing processes and fibrotic responses are characterized by type 2-like environment governed by the presence of IL-4, IL-13, ILC2, Th2-like T cells, and M2 macrophages—also named alternatively activated macrophages—all discussed in following paragraphs [87–89]. Very likely, type 2 environment plays an important role in SSc, particularly in skin fibrosis [90]. Specificities related to organs and tissues undergoing fibrotic changes are being unraveled by “omics” studies at single-cell level and are revealing the presence of rare cell types with specific phenotypic and functional characteristics [91]. Within this framework, the response to the master pro-fibrotic cytokine TGF-β is thought to be dysregulated in SSc. TGF-β is considered to be, at least partly, responsible for the fibrotic disease component. TGF-β induces fibroblast migration, proliferation, and differentiation and enhances ECM production components including various collagens [92, 93]. TGF-β has pleiotropic functions, is produced by many cell types in association with latency-associated peptide (LAP), interacts with the ECM, and requires processing to become biologically active. It binds to a heterodimeric receptor which intracellular signal is mediated by canonical SMAD signaling and complex, additional non-canonical pathways. The activity of TGF-β is tightly regulated at several levels including the availability of the biological active form, receptor binding, and most importantly the intracellular signaling pathway level which offers potential targets of treatment [94]. Connective tissue growth factor (CTGF) also known as CCN2 appears to be a necessary cofactor for TGF-β to activate or sustain extracellular matrix (ECM) production in both healthy and disease states [95]. Platelet-derived growth factor (PDGF), IL-6, Wnt/β-catenin (Wnt: Wingless and Int), and hedgehog signalling are some of the other important components of the profibrotic milieu [96]. As a word of caution, our understanding of the main forces involved in fibrosis, namely in SSc skin fibrosis, remains imprecisely defined. When submitting skin biopsies from the involved SSc skin to unbiased gene expression studies, heterogeneous results were obtained across skin samples. Patient samples were grouped according to the main gene expressed into an “inflammatory,” “fibroproliferative,” “limited,” or “normal-like” gene-signature [97–100]. These results point to heterogeneous mechanisms leading to skin fibrosis which do not match, or match only partially, to clinical classifications and histories.

### Myofibroblast

**Fig. 4:**
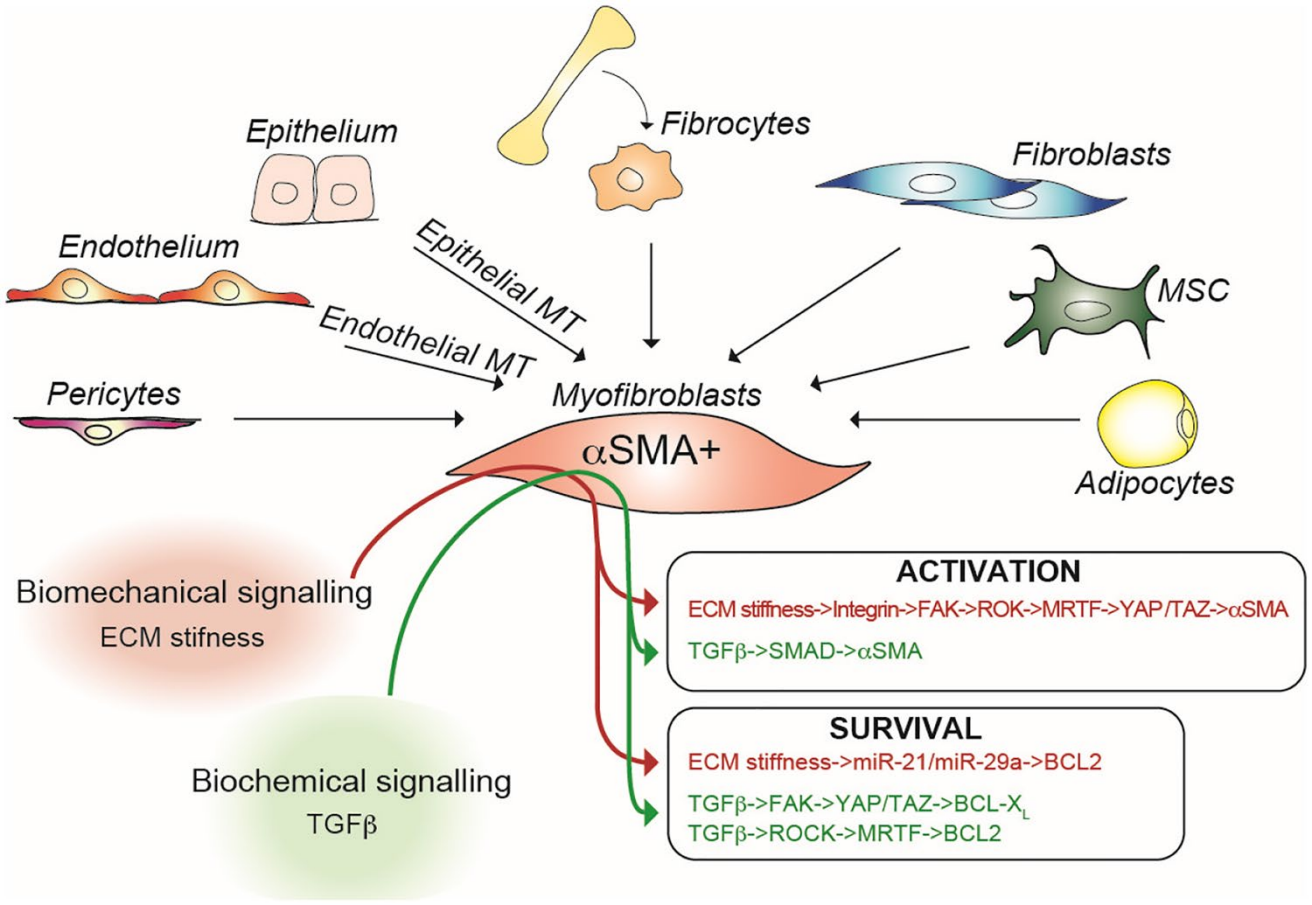
Myofibroblasts and their centrality in the development of fibrosis in SSc. Depicted are the cells potentially giving origin to myofibroblasts, as well as the main signals involved in their activation and survival. αSMA, alpha smooth muscle actin; BCL, B-cell lymphoma; ECM, extracellular matrix; FAK, focal adhesion kinase; MRTF, myocardin-related transcription factors; ROCK, Rho-associated creatinine kinase; SMAD, small mothers against decapentaplegic; TAZ, transcriptional co-activator with PDZ-binding motif; TGF-β, transforming growth factor-beta; YAP, Yes kinase-associated protein

Large agreement identifies in myofibroblasts the professional cells involved in the enhanced ECM deposition occurring during fibrosis development. At variance of what occurs during wound healing, in fibrotic processes myofibroblasts after having been activated or transdifferentiated do not stop producing ECM, possibly because they become resistant to apoptosis-inducing signals [101] (Fig. 4). Most recently, it has been proposed that myofibroblasts are characterized by increased levels of pro-apoptotic intracellular mediators, compensated by even higher levels of anti-apoptotic intracellular mediators. Among the most likely mechanisms responsible for such altered balance set-point, stiffness of tissue undergoing fibrosis transduced by mechano-sensors to myo-fibroblasts appears to play an important role [102]. It is unlikely that fibroblasts autonomously initiate the fibrotic response, however with time they may become independent from initiating stimuli. As an example, ECM stiffness enhances the release of latency associated peptide (LAP), followed by activation of transforming growth factor-β (TGF-β) by α_v_-integrins which then favors further ECM deposition [103]. Similarly, fibronectin extracellular domain A (FN^EDA^), expressed in high amounts in involved SSc skin, was shown to bind TLR4 and enhance collagen production in an *in vivo* murine model of scleroderma. FN^EDA^ production is induced by TGF-β and simultaneously enhances TGF-β production by fibroblasts thus providing a positive feedback loop potentially able to maintain in an autonomous manner sustained fibroblast activation [104]. It is also likely that many stimuli of different origin may converge on fibroblasts which response is then monomorphic [96]. It is however important to stress that several subpopulations of fibroblasts have been documented which may play distinct and dynamic roles in tissue homeostasis and fibrosis [105–107]. In this respect, myofibroblasts are capable of contractile properties and are considered professional ECM producers [88, 108, 109]. Their origin is debated and has been ascribed variably at resident fibroblasts, at circulating fibrocytes (cells of hematopoietic origin with mesenchymal properties including the capacity to produce collagen), at smooth muscle cells, at epithelial cells undergoing mesenchymal transition, or similarly at endothelial cells undergoing mesenchymal transition (Fig. 4) [110]. Cell fate tracing *in vivo* experiments has however pointed to a larger contribution of pericyte transdifferentiation for the generation of myofibroblasts. Pericytes are naturally endowed with contractile properties and acquire the capacity to produce ECM components upon migration into tissues undergoing fibrosis [88]. Thus, migration, proliferation, differentiation of fibroblasts and the relationship they establish with ECM and tissue physical properties via mechanosensors are key to fibrosis development and persistence [91] (Fig. 4).

### Keratinocytes in SSc

**Fig. 5:**
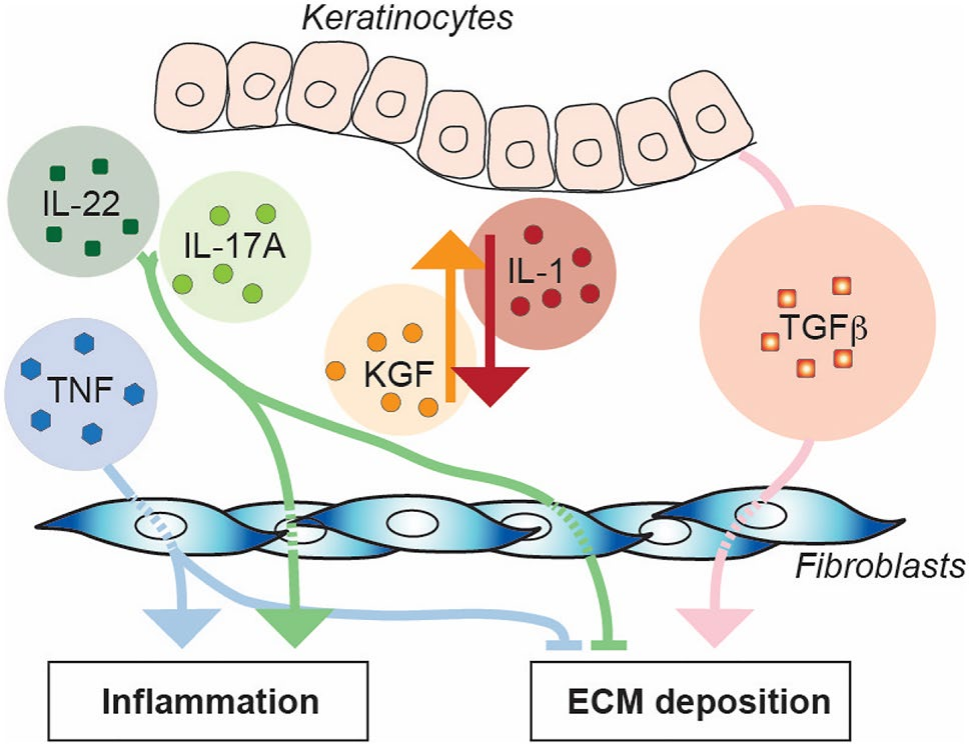
Altered cross-talk between keratinocytes and dermal fibroblasts in SSc. The homeostatic relationship between epidermis and dermis includes reciprocal signaling here represented by IL-1 produced by keratinocytes and KGF by fibroblasts. Cytokines dysregulated in SSc alter this cross-talk and variably affect the inflammatory and ECM deposition properties of dermal fibroblasts. ECM, extracellular matrix; KGF, keratinocyte growth factor; IL, interleukin; TGF-β, transforming growth factor beta; TNF, tumor necrosis factor

The epidermis and in particular keratinocytes participate to dermal homeostasis by releasing factors that target dermal fibroblast. Reciprocally, keratinocytes respond to soluble mediators released by dermal fibroblasts [111]. Thus, it is not surprising that SSc epidermis presents a variety of abnormalities including altered differentiation, active TGF-β signaling, increased production of antimicrobial peptides, with DAMP properties, enhanced capacity to stimulate lattice contraction and inflammatory responses in dermal fibroblasts [112–117]. Furthermore, epithelial deficiency of the transcription factor Fli1 in mice is sufficient to induce a SSc-like phenotype, including fibrosis and systemic autoimmunity [118]. Within this framework, SSc keratinocytes, engineered epidermal equivalents, or organotypic full skin cultures were shown to respond to cytokines which levels are increased in SSc by further modulating dermal fibroblast responses. IL-17A and IL-22 in conjunction with TNF were shown to enhance inflammatory dermal responses and in particular IL-17A was shown to counteract, at least partially, the profibrotic activity of TGF-β by modulating the Wnt/β-catenin signals [119, 120]. These are examples of intercellular circuitries potentially aiming at reducing fibrosis still participating to inflammation in SSc altered homeostatic conditions (Fig. 5).

## Autoimmunity and Inflammation in SSc

### Immunological Tolerance Defects in SSc

SSc is considered a systemic autoimmune disorder, characterized by the presence of autoantibodies directed against ubiquitous (mostly nuclear auto-antigens) as well as cell-specific autoantigens. Similarly, autoreactive T cells have been demonstrated recognizing epitopes of the ubiquitous autoantigens topoisomerase-I (topo-I) and RNA polymerase III (RNApol-III) [121, 122]. Indirect proof of autoimmunity from the T cell point of view is the oligoclonal distribution of TcRs of T cells retrieved from SSc blood, skin, and lung, suggestive of an (auto)antigen-driven clonal expansion [67, 123]. In addition, the strong association of SSc with specific HLA alleles supports an immune component in the pathogenesis of SSc. Consistent with this view the survival advantage provided by profound pharmacological immunosuppression rescued by autologous hematopoietic stem cell transplantation in severe SSc [124–126]. However, standard immunosuppression has limited efficacy in SSc when compared to other autoimmune systemic disorders. Thus, while defective immune tolerance has a role in SSc pathogenesis, possibly in very initial events, important additional singularities characterize SSc leading to sustained vasculopathy and fibrosis. Interestingly, the presence of autoAb directed against distinct ubiquitous autoantigens is usually mutually exclusive and clinical manifestations segregate with the type of autoAb, which supports a pathogenic link between autoAb specificities and clinical manifestations. However, there is no experimental proof of such a link and for the moment being autoAb directed against ubiquitous antigens are considered only as epiphenomena, tough clinically useful as biomarkers.

Autoimmunity in SSc requires both innate and adaptive immune responses at humoral and cellular levels which participate to disease initiation under the influence of some of the triggers previously mentioned. While no animal model faithfully reproduces all the clinical and biological features of SSc, it is worth to stress that repeated mice immunization with T and B cell autoantigen Topo-I and concomitant stimulation of the innate immune response by complete Freund adjuvant results in a disease characterized by skin and lung fibrosis and autoimmunity in C57Bl/6 mice [127]. No such results were obtained in autoimmune prone mice when immunized with Topo-I in the absence of solid innate immunity activation [128]. The evidence thus generated strongly support the need of multiple, sustained hits to break tolerance and initiate processes leading to SSc. In humans, molecular mimicry is potentially implicated in tolerance breakdown. For instance, experimental evidence suggestive for SSc having a paraneoplastic origin has been provided in association with mutated RNA pol III antigen [38]. Similarly, cross-reactive antibodies between CMV UL94 antigen and endothelial cells have been documented [32].

### Innate Immune Cells, Soluble Products, and Receptors in SSc

**Fig. 6:**
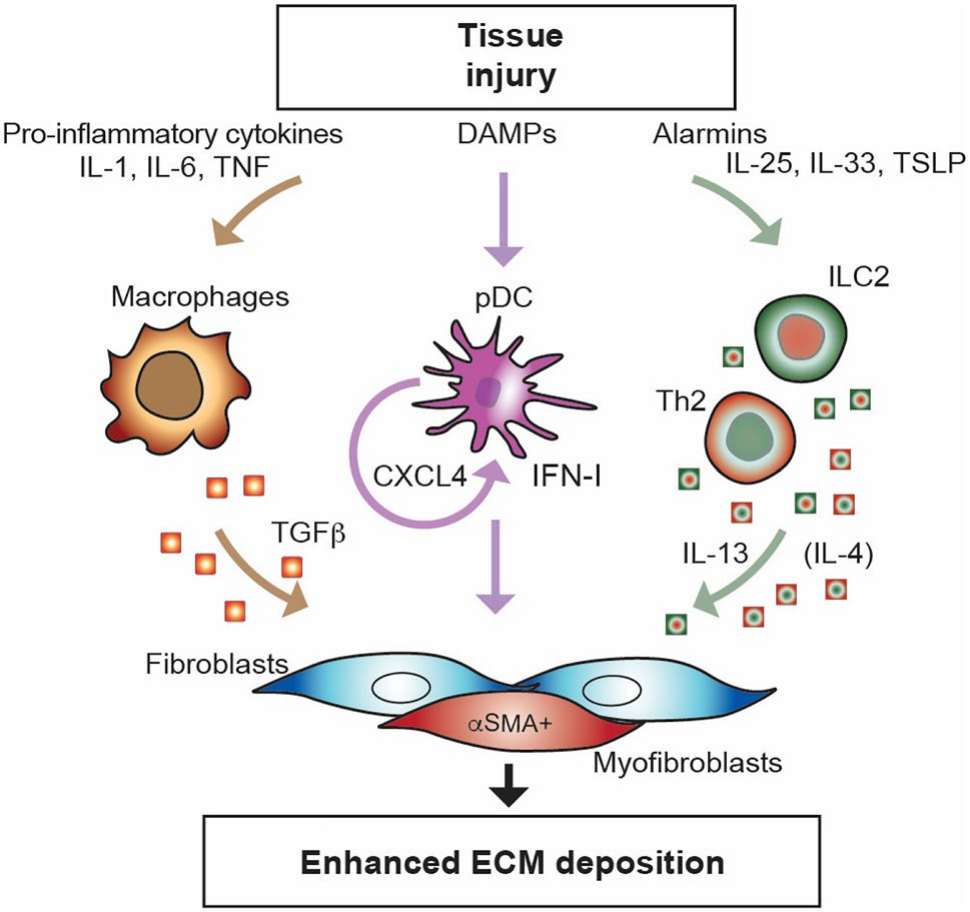
Contribution of cells and soluble products of the innate immune system to enhanced ECM deposition. Parallel, not mutually exclusive pathways involving cells of the innate immune system and their soluble products, converge on fibroblasts enhancing their ECM synthetic capacity. CXCL, chemokine containing the CXC motif; DAMP, danger associated molecular patterns; IFN-I, type I interferon; IL, interleukin; ILC, innate lymphoid cell; pDC, plasmacytoid dendritic cell; TGF-β, transforming growth factor-beta; Th2, type 2 T helper cell; TNF, tumor necrosis factor; TSLP, thymic stromal lympopoietin

All innate immune cells and their capacity to be activated in the one hand by pathogen (PAMP) or danger due to tissue damage (DAMP) molecular patterns via PRRs and on the other hand by soluble mediators of inflammation (cytokines, chemokines, lipidic mediators, NO, etc.) participate to SSc pathogenesis. Of interest, PRR are not only expressed by innate immune cells but also by stromal cells including fibroblasts and endothelial cells, where they are thought to play a substantial role. Similarly, stromal cells can produce and respond to soluble mediators of inflammation. Thus, an intricate web of signals to cells and responses by cells constitute the inflammatory network that in SSc extends well beyond the “classical” components of the immune system. Deciphering this network will potentially provide hierarchically important nodes as target for therapeutic interventions. Broadly speaking, by sensing altered homeostasis and tissue damage, cells of the innate immune system may contribute in many ways to initiation and amplification of inflammatory events leading to fibrosis [91]. On the one hand, the release by cells submitted to stressful signals of pro-inflammatory cytokines, including IL-1, tumor necrosis factor (TNF), and IL-6 may turn on macrophages which may initiate TGF-β release and activation. In the other hand alarmins release, including IL-33, IL-25 (also known as IL-17E), and thymic stroma lymphopoietin (TSLP), may activate type 2 innate lymphoid cells (ILC2), which participate to Th2-like T cell responses and enhance the production of IL-4 and IL-13 which directly and indirectly participate to enhanced ECM deposition. Furthermore, myeloid dendritic cells (mDCs) and plasmacytoid DC (pDC) contribute to generate the fibroproliferative milieu by releasing type I interferon (Fig. 6).

### PRR and SSc

Given their central role in sensing danger, whether due to infectious agents or tissue damage, PRR undoubtedly plays a major role in SSc [129]. The contribution of PRR to SSc pathogenesis has received increasing attention in two distinct directions: the role of PRR in the production of type I interferons (IFN-I) or other pro-inflammatory cytokines and the contribution of TLRs in activating mesenchymal cells, in particular fibroblasts. Here follow a few examples. TLR4, which expression is increased on SSc fibroblasts [130], mediates chronic fibroblast activation by sensing FN^EDA^ [104]. Consistently with a role in fibroblast activation, an amelioration of tissue fibrosis was observed in TLR4 knockout in murine models of systemic sclerosis [131]. TLR8, expressed in monocytes, may mediate their transdifferentiation in fibroblasts, potentially responding to lytic EBV infection [132]. SSc monocytes upon TLR8 activation by ssRNA (and to a lesser extent by LPS/TLR4) produce enhanced levels of tissue inhibitor of matrix metalloproteinase (TIMP)-1 [133]. TLR8, paradoxically expressed in SSc pDC, plays a role in IFN-I production [134]. Always in pDC, TLR7 and TLR9 play a role in sensing DNA or RNA shuttled by autoantibodies via Fc-gamma receptors or by CXCL4 into the endosomal compartment thus also participating to enhanced levels of IFN-I in SSc [135, 136]. In addition to TLR9, cytosol-located GAS-STING activation by mitochondrial DNA—which concentration is increased in SSc plasma—was shown to be positively associated with IFN-I and IL-6 expression and SSc-ILD progression [137].

In SSc, circulating *monocytes* and tissue-resident *macrophages*, potentially under the influence of type 2 cytokines (IL-4, IL-13), appear to preferentially express CD163 and CD204 and promote fibrogenesis by increasing the production of TGF-β. They are involved also in the production of a large variety of other inflammatory mediators including chemokines, cytokines, matrix metalloproteinases (MMPs) and their inhibitors (TIMPs), which composition and role may depend on timing and localization. They are likely involved both in vasculopathy as well as in fibrosis and they may play a role in the perpetuation of the disease having pro-reparative properties inefficiently terminated [138]. In an experimental murine model of SSc, it was shown that epigenetic modifications of macrophages (trained immunity) induced by activation in the one hand with low-dose lipopolysaccharide (LPS), on the other hand by BCG (Bacillus Calmette Guérin) could deeply influence the fibrotic response with reduced or enhanced fibrosis, respectively [139]. Thus, macrophages sensing pro-fibrotic cues may propagate or amplify tissue fibrosis. mDCs, beside their role as antigen-presenting cells (APCs) may play relevant inflammatory functions in SSc [138]. Tissue-resident *plasmacytoid DC* (pDC) also may play a substantial role. pDC were shown to respond to CXCL4 (CXC chemokine ligand 4, also known as platelet factor 4, PF4) which levels are highly increased in SSc sera [140] and forms complexes with DNA [136]. These complexes are shuttled into the endosomal compartment where by interacting with TLR8 or TLR9 favor the production of IFN-I, highly increased in about 50% of SSc individuals [134, 136]. The relevance of these findings was highlighted by the prevention of skin inflammation and fibrosis in xenotransplant human-mouse model of scleroderma by targeting human pDC [141]. The most recently described in the innate cell family are the *innate lymphoid cells* (ILCs). They are endowed with the capacity to rapidly produce polarized subsets of cytokines under the control of differentially expressed master transcription factors. They are activated by PRR ligation and are fast producers of cytokines. Relatively little is known yet about ILCs in SSc; however, evidence points to an expansion of ILC2 (producing IL-4/IL-13) in the blood and in the skin. Thus, they may contribute to a dysregulated environment favoring fibrosis [142]. ILC2, in particular the KLRG1neg ILC2 subset numbers appear to be increased in SSc skin correlating with the extent of skin fibrosis. Of note, TGFβ favors the expansion of the KLRG1neg ILC2 subset and simultaneously decreases their production of IL10, which regulates negatively collagen production by dermal fibroblasts [143]. This example highlights the intricate relationship in the cytokine network that portends enhanced deposition of ECM.

### ROS in SSc

An imbalance between oxidant and anti-oxidant states is observed in SSc, with increase in the blood of oxidative stress biomarkers such as malondialdehyde (MDA—a marker of lipid peroxydation), nitric oxide and endogenous nitric oxide inhibitor asymmetric dimethylarginine (ADMA) and decreased anti-oxidative biomarkers, such as superoxide dismutase and vitamin C [144]. ROS may impact on monocyte/macrophages polarization favoring M2-like differentiation [145]. ROS participate to fibroblasts activation triggering the production of pro-inflammatory cytokines such as IL-1β and fibroblasts from SSc are a potent source of ROS through an up-regulation of NOX-2 and NOX-4 [146]. Further, inflammasome, in particular NLR family pyrin domain containing 3 (NLRP3) inflammasome is thought to be involved in fibroblasts [147, 148], endothelial cells, and macrophages activation in SSc [149]. NLRP3 expression is increased in SSc skin and NLRP3-deficient mice are resistant to bleomycin-induced fibrosis. It is possible that oxidative stress could participate to NLRP3 activation [10].

## Adaptive Immunity in SSc

### T Cells in SSc Skin

**Fig. 7:**
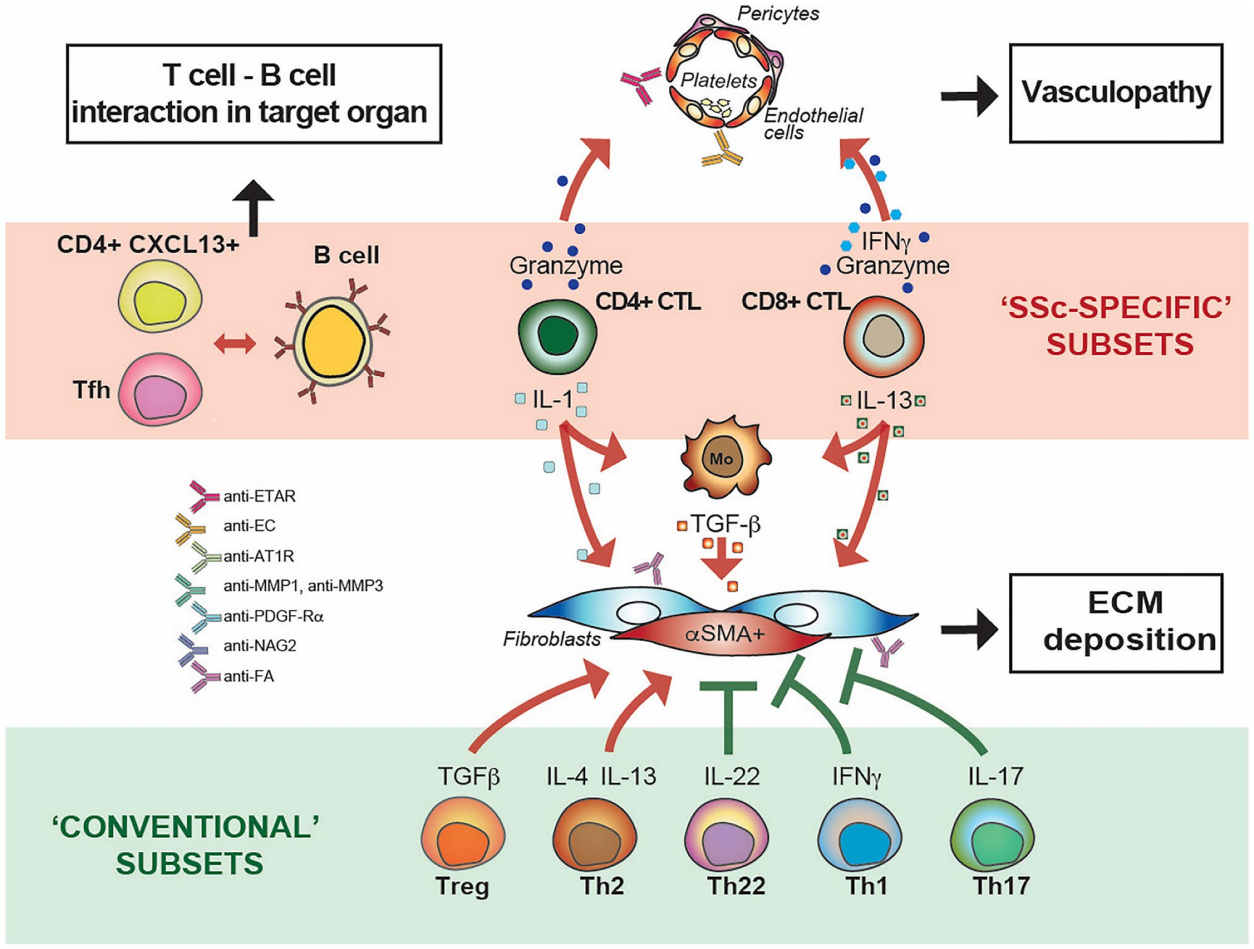
Adaptive immune responses and their roles in SSc. T cells, B cells, and their products contribute to both enhanced ECM deposition and vasculopathy. This schematic representation highlights the characteristics of conventionally defined as well as of SSc-restricted T cell subsets. They may have enhancing or inhibitory functions (blunted heads: inhibitory function; arrowhead: enhancing function). T-B cell interactions are important for both the generation of agonist/antagonist autoAb and tissue damage. CD, cluster of differentiation; CXCL, chemokine containing the CXC motif; IFN-γ, interferon-gamma; IL, interleukin; T_FH_, T follicular helper cell; Th, T helper cell. Autoantibody specificities: AFA, anti-fibroblast; AT1R, angiotensin-II receptor; ECA, endothelial cell; ETAR, endothein-1 receptor A; MMP, matrix metalloproteinase; NAG2, also known as transmembrane 4 superfamily member 7; PDGF-Rα, platelet-derived growth factor receptor-alpha; UL-94, gene coding for the cytomegalovirus (CMV) cytoplasmic envelopment protein 2

Compared to healthy skin, T cells are abundant in involved SSc more so early in disease course and active collagen synthesis is more pronounced around inflammatory infiltrates [150, 151]. T cell effector functions are highly heterogenous and differentially affect fibrosis and vasculopathy. Within the *adagio* that type 2 responses favor repair and fibrosis, Th2 cells (CD4+T cells producing IL-4 and IL-13) as well as Tc2 (CD8 T cells producing IL-13), in conjunction with the previously mentioned ILC2 may be actively involved in enhancing ECM deposition, since both IL-4 and IL-13 can directly enhance collagen production by fibroblasts [90, 152]. Further, IL-13 may enhance the production of TGF-β by macrophages thus indirectly enhancing ECM deposition [153]. In addition, SSc-skin infiltrating Treg cells, under the influence of IL-33, may become Th2-like effectors and release profibrotic cytokines contributing to enhanced ECM deposition (Fig. 7). However, Th1, Th17, and Th22 cells may also be enriched in SSc skin where they potentially participate to inflammation simultaneously, and most importantly, opposing fibrosis [119, 120, 154, 155]. From another angle, CD4+ and CD8+ T cells with cytolytic potential present in SSc skin may participate to vasculopathy by enhancing endothelial cell apoptosis [67]. The presence of high-affinity, isotype switched, autoantibodies characteristic of SSc is further strong evidence for the role and contribution of T helper cells, in particular of follicular T cells (T_FH_) in SSc. Indeed, T_FH_ cells are increased in SSc peripheral blood and in the skin, they present an activated phenotype, increased capacity to produce IL-21, and higher capacity to stimulate the differentiation of CD19+CD27+CD38hi B cells and their secretion of IgG and IgM through the IL-21 pathway than healthy controls. In experimental SSc, the blockade of IL-21 or of inducible T cell co-stimulator ICOS (expressed by T_FH_) resulted in decreased skin fibrosis establishing a link between T_FH_ cells and an immune-mediated fibrotic reaction [156, 157]. Finally, a study based on single-cell RNAseq has identified eight distinct T cell clusters, of which one uniquely present in SSc skin 10.1136/annrheumdis-2021-220209. This CD4+ T cell subset is characterized by the expression of CXCL13 and IL-21 in addition to an T_FH_-like gene expression signature and that appears to be poised to promote B-cell responses within the inflamed skin of patients. Thus, the composite picture provided by studies focusing on T cells in SSc is highly complex which may depend on the timing along disease course in which the study is made, with a relative predominance of type 2 responses early and of type 1 (IFN-γ) and 17 later in disease course, when fibrosis tends to decrease, at least in the skin (Fig. 7).

### B Cells and Humoral Immunity in SSc

B cells participate deeply to SSc pathogenic events both as precursors of autoAb producing cells and as inflammatory cells infiltrating tissues undergoing fibrosis, namely the skin and the lung [97, 158] where they release cytokines and may influence the behavior of fibroblasts and other mesenchymal cells [159] (Fig. 7). Reduced numbers of the Breg subset with decreased production of IL-10 have also been documented and may participate to the dysregulated regulatory network in SSc [160].

Beyond autoAb directed against nuclear antigens characteristic of SSc, cell-specific autoAb may very well be pathologically relevant. Examples are autoAb directed against the receptor A of endothelin-1 (ETAR) or the angiotensin-II type 1 receptor (AT1R), which were shown to affect several processes ranging from production of collagen by skin fibroblasts to angiogenesis modulation [7]. Antagonist autoAb directed against MMP1 and MMP3 were described to block the enzymatic activity of these proteins, thus reducing the digestion of matrix [161, 162]. In the other hand, autoAb with relevant agonist properties are those directed against the PDGFRα with induction of the ROS-ERK1/2-Ha-Ras loop and increased collagen gene transcription in human fibroblasts in vitro and in vivo in a humanized mouse model of skin fibrosis [163, 164]. These autoAb can be considered pathogenic and participate to disease progression in conjunction with those directed against endothelial cells [64].

### Microbiome and SSc

No doubt that microbiome influences deeply the immune response and this in two main ways [165] first, representing an antigenic challenge with whom the immune system needs to cope, mostly by establishing tolerance via different mechanisms but also generating specific innate and adaptive responses; second, by enforcing nutritional and metabolic cues that influence the immune response, beside the behavior of other host systems. Dysbiosis is a modification of microbiota with relevant immunological and metabolic consequences. Of interest, main organs affected in SSc are the skin, the lung and the gut, which are barrier organs in which the microbiota resides. SSc-associated dysbiosis has been documented in the skin with decreased lipophilic taxa and a marked increase in a wide range of gram-negative taxa [166]. In the gut, a distinct microbial signature in SSc patients compared with healthy controls has been documented [167, 168] with indications for a reduction in protective butyrate-producing bacteria and by an increase in proinflammatory noxious genera, especially *Desulfovibrio* [169]. Similar findings were reported in gut microbiomes of IgG4-related disease and SSc patients showing increase in opportunistic pathogenic *Clostridium* and *Streptococcus* species, while butyrate-producing species were depleted. Interestingly, the gut microbiomes of IgG4-RD and SSc showed signatures similar to those found in multiple sclerosis and rheumatoid arthritis, but not those found in inflammatory bowel diseases where the most differentially abundant taxa are facultative anaerobes [170]. Thus, it is likely that the dysbiosis may influence disease initiation and disease evolution. At this time point, however, whether the microbiome alterations documented in SSc are primary or secondary to organ pathology and/or medication use is not yet established.

## System Biology Approaches to Decipher SSc

Within the last decade or so, we have witnessed the increased application of techniques based on the unbiased identification of gene expressed in SSc affected organs, particularly but not exclusively the skin and the peripheral blood, and more recently single cell RNAseq, that exponentially increase the amount of information on cellular and tissue alterations characterizing SSc. Additionally, multi-“omics” approaches exploring metabolism, epigenetic modifications, phenotypes, etc. further contribute novel information. The wealth of data is then submitted to sophisticated analysis based on complex algorithms aiming at reducing the catalogued data to integrated dimensions that are comprehensible, simultaneously providing new understanding or novel perspectives for old knowledge. This type of studies should provide a wider conceptual framework to better understand SSc physiopathology.

Historically, the Whitfield group published the first gene array study on skin biopsies. Expressed genes differentiated SSc from healthy controls and were similarly expressed in involved and not involved skin [97]. Further analysis based on genes expressed in skin provided evidence for the existence of intrinsic SSc subsets named “inflammatory”, “fibroproliferative,” “limited,” or “normal-like” [171]. Active immune and defense responses were associated with the inflammatory subset; proliferation and cell cycle programs with the fibroproliferative subset; and the normal-like subset was associated with a distinct lack of inflammatory signature coupled with fatty-acid metabolism. The limited subset showed deregulation of pathways associated with cell adhesion, cardiovascular system development, ECM, and immune and inflammatory responses [172]. According to these authors, the SSc intrinsic subsets were relatively stable throughout disease course and unlikely to change over time [98]. To identify genes co-expressed across various cohorts, consensus clustering analysis led to the identification of conserved genes and networks common to distinct subsets [173]. The connected gene-gene networks included the terms: “adaptive immunity,” “interferon,” “M2 macrophages,” “ECM,” and “proliferation.” A meta-analysis of genes expressed in multiple end-target organs including the skin, the lung, the esophagus and the peripheral blood provided evidence for the occurrence across organs of the intrinsic subsets, pointing to the existence of pro-fibrotic macrophages in multiple tissues [174].

Similar, but not identical, gene signatures were found by other authors with the identification of two prominent transcriptomes in SSc skin: named the “keratin” and “fibroinflammatory” signatures. The first associated with shorter disease duration the second with diffuse cutaneous involvement and a higher modified Rodnan skin score (mRSS). A subgroup of patients with significantly longer disease duration had a normal-like transcript pattern [175]. Further data from the same group reinforced the concept that gene expressed in early disease had higher adaptive immune cell signatures than in later disease, while fibroblast and macrophage cell type signatures were associated with higher mRSS. Of further interest, the immune cell signatures correlated with the rate of skin thickness progression prior to, but not after, biopsy [151]. Overall, these results support the concept that the pathological processes characterizing SSc may be different during the disease evolution and enrich our understanding by subgrouping patients on the basis of preferential gene expression in target organs.

In another study, by generating a normalized catalog of differentially expressed genes (DEGs) from 344 skin samples of 173 patients and submitting DEG to pathway analysis, patients with SSc were grouped into four distinct clusters that differed in activation levels of SSc-relevant signaling pathways. In this analysis, the phosphoinositide-3-kinase protein kinase B (PI3K-Akt) signaling pathway showed the closest correlation and temporal association to mRSS. Interestingly, the inflammatory subtype was related to significant improvement in skin fibrosis at follow-up in the absence of specific treatment [176].

The identification of 415 DEG in skin differentiating SSc from HC allowed the generation of a score, named 4S, correlating with mRSS and potentially useful to predict response to treatment [99].

The identification of genes defining intrinsic SSc subsets provides ground for applying precision medicine in therapeutic approaches. Retrospective analysis of data generated during therapeutic trials has indeed offered elements supportive for responses to therapeutic agents depending on the intrinsic subset of the treated individual. Thus, agents like mycophenolate mofetil or abatacept targeting the immune response may be efficacious in the inflammatory subset [177–179], while “anti-fibrotic” agents may be more efficacious for the fibroproliferative subset [180, 181]. Potentially surprising, in a *post hoc* analysis, individuals belonging to the fibroproliferative subset presented a significant advantage in event free survival when undergoing hematopoietic stem cell transplantation (HSCT) compared to individuals treated with cyclophosphamide in the SCOT trial [182]. HSCT tended to confer an advantage over cyclophosphamide to individuals of the inflammatory subset, with no differences between treatment arms for individuals belonging to the normal-like subset. Limiting factor in the interpretation of these data was the low number of samples and individual trajectories available for these analyses; that however could provide novel dimensions in the selection of patients included in clinical trial beside classic clinical classification.

To summarize pathophysiological information gathered until now by using big data and unbiased methods to identify SSc specificities, it appears that SSc heterogeneity extends beyond and does not overlap with classical clinical and serological parameters, that predominant gene signatures—intrinsic subsets—differ among SSc individuals and tend to persist during disease evolution, with however enrichment for immune response genes earlier, and macrophage—fibroblast gene later in disease course in severe cases. Not yet confirmed in prospective studies, responses to therapeutic approaches may differ among patient subsets according to the mechanism of the therapeutic agent assessed.

## Conclusions and Perspectives

SSc represents a major challenge for our understating of physio-pathological processes leading to disease state and disease progression. The heterogeneity in SSc clinical manifestations influences disease identification and classification and, to a certain extent, our approach to medical management. However, the subtle mechanisms underpinning clinical heterogeneity are, by far, poorly understood. Major progress in our understanding, based on increasingly more precise identification of cell types and intercellular signaling as well as of intracellular molecular cues at play in SSc physio-pathology has spurred enthusiasm in the scientific community and has led to the recent approval of therapeutic agents that may alter the disease course. We should admit, however, that the pace of improvement is slow and a major gap still exist between scientific advancement and clinical application. We believe that the integration of “omics” approaches to sophisticated system biology analyses will contribute to the refinement of our understanding and should be intensively applied, particularly in controlled clinical trials. By employing these methods, the comparisons in tissue responses between placebo and active arms in well characterized patient populations should provide interesting new information on mechanisms at play in subsets of patients and their deviation under the pressure of therapeutic agents. In view of SSc clinical heterogeneity, possibly linked to heterogeneity in pathogenic mechanisms, it is unlikely that the responses to a given agent will be homogeneous. We believe that differences in responses within supposedly homogenous subsets of patients will be extremely informative from the pathogenic point of view and will provide substantial advancement. In this perspective, we propose that small rather than large trials, in which deep “omics” will be applied to extremely selected group of patients will provide relevant information. To overcome the constrains linked to the rarity of the disease, these trials should be conducted across multiple, well-coordinated, integrated, and equipped centers. Trials in which mechanisms will be primary outcomes will provide solid ground for solid therapeutic secondary outcomes.
